# Newly diagnosed chronic granulomatous disease in a 44 year old male presenting with recurrent groin cellulitis and colitis

**DOI:** 10.1186/1710-1492-9-9

**Published:** 2013-03-06

**Authors:** Arthur G Chung, Michael M Cyr, Anne K Ellis

**Affiliations:** 1 West Oak Medical Clinic, Oakville, ON, Canada; 2 Division of Clinical Immunolgoy & Allergy, McMaster University, Hamilton, ON, Canada; 3 Division of Allergy & Immunology, Department of Medicine, Queen’s University, Kingston, ON, Canada; 4 Allergy Research Unit, Kingston General Hospital, Queen’s University, 76 Stuart Street, Kingston, ON K7L 2V7, Canada

## Background

Chronic Granulomatous Disease (CGD) is an inherited deficiency which results from the absence or dysfunction of nicotinamide adenine dinucleotide phosphate (NADPH) oxidase subunits in phagocytic cells. This condition is usually diagnosed in early childhood and presents with recurrent infections at epithelial surfaces such as the skin, lungs, and gut. We present a case of CGD which was diagnosed late in adulthood.

## Case presentation

A forty-four year old male was admitted to hospital with fever, testicular pain and an acute onset of swelling of his penis and scrotum.

His past history was significant for Crohn’s disease diagnosed at age sixteen; stable on treatment with mesalamine (Asacol®). He also had recurrent soft tissue infections of his penis and/or scrotum since childhood, approximately 2 or 3 in total, to his recollection. There was no history of abscesses or sexually transmitted infections. However, the patient did complain of frequent upper respiratory tract infections each winter. He had no family history of primary immunodeficiency disease.

During admission, he was assessed by urology and general surgery. Both confirmed the absence of any anatomic abnormalities. An immunologist suggested work up for immunodeficiency. Basic laboratory investigations were normal, with a white blood cell count of 7.8 × 10^9^ L. C-reactive protein was elevated at 69.7 mg/L. Both his rheumatoid factor and HIV testing were negative. Serum levels of IgA, IgM and IgG levels were all within normal limits (Table 1 reports full details of his immunology investigations) Blood cultures were negative. A CT scan of the chest was normal. CT scan of the abdomen and pelvis revealed no subcutaneous air to suggest a necrotizing infection.

**Table 1:** Results of Immunologic Investigations

**Parameter**	**Value**	**Reference range**
WBC	7.8 × 10^9^/L	4.0 – 11.0 × 10^9^/L
CD19 +	11%	4 – 26%
CD4+	0.87 × 10^9^/L	0.50 – 1.75 × 10^9^/L
CD8+	0.32 × 10^9^/L	0.15 – 0.84 × 10^9^/L
IgA	3.53 g/L	0.70 – 3.52 g/L
IgG	12.50 g/L	6.35 – 14.65 g/L
IgM	1.72 g/L	0.41 – 2.07 g/L
CGD Test	Absence of Oxidative Burst Response	

Quantitative dihydrorhodamine (DHR)123 flow cytometry assay revealed the complete absence of an oxidative burst response; this was confirmed three times. This was suggestive of a neutrophil NADPH oxidase deficiency associated with CGD.

The patient was treated with intravenous cefazolin and metronidazole and discharged home on clindamycin. His cellulitis resolved within weeks, but he experienced a recurrence approximately 6 months later. He was started on trimethoprim-sulfamethoxazole (TMP-SMX), amoxicillin, and itraconazole as prophylactic therapy to prevent future infections. He was assessed by the Infectious Disease service, who simplified his prophylactic regimen to TMP-SMX 160/800 q Mon/Wed/Fri and Penicillin V K 300mg OD based on risk assessment and previous infective patterns. Interferon-gamma was considered, but based on restricted availability at the time and his relatively stable clinical course to date, we elected to pursue antibiotic prophylaxis and active surveillance alone.

Genetic testing, performed by the Hospital for Sick Children in Toronto, confirmed a diagnosis of CGD and revealed a homozygous mutation in the NCFI gene (P47). It was recommended that directly family members receive DHR flow cytometry screening but this was declined.

## Discussion

CGD is a rare disorder with an approximate incidence of 1 in 200,000 births [1]. A mutation in one of four protein subunits of the NADPH oxidase complex (gp91-, p22-, p47-, p67-phox) result in CGD. The NADPH oxidase enzyme complex is responsible for production of superoxide anions required for a cellular respiratory burst. Patients deficient in the NADPH oxidase enzyme become susceptible to recurrent life-threatening infections by a spectrum of fungi and bacteria, most commonly Aspergillus and Staphyloccocus spp [1]. There are 410 known mutations of the 4 affected genes with 76% related to a mutation of gp91-phox, the x-linked recessive form. The p47-phox mutation occurs in 18%, while the p67 and p22 mutations are less common (4 and 3%, respectively) [1].

The most common infections in CGD include pneumonia, abscess and suppurative adenitis (79%, 68%, and 53% respectively), while cellulitis occurs in only 10% [1]. CGD can also present with a granulomatous colitis mimicking Crohn’s disease in 17% [1].

CGD patients can be vaccinated with measles, varicella and annual influenzae, but BCG should be avoided because of the risk of developing disseminated BCG-osis [2]. Prophylactic antibiotics and anti-fungal agents such as trimethoprim-sulfamethoxazole and itraconazole have been found in small studies to reduce rates of infections, surgical interventions and hospitalization [3,4]. Prophylactic IFN-γ given thrice weekly may reduce the risk of serious infections [5]. Bone marrow transplantation provides definitive treatment but is reserved for the most severe cases and gene therapy with retroviral vectors may be a promising solution in the future [6].

CGD is usually diagnosed in childhood. The mean age of diagnosis for the x-linked recessive form is 3 years of age while the autosomal recessive forms are diagnosed at 7.8 years of age [1]. A small number of CGD cases have been diagnosed in adulthood beyond the fourth decade [7-10]; the oldest case report identified in the English language literature was of a male diagnosed at 69 years of age [11].

## Conclusions

In summary, we present a patient who was diagnosed with CGD in adulthood with manifestations limited to recurrent scrotal cellulitis and a granulomatous colitis, which was initially misdiagnosed as Crohn’s disease. Adults with a history of recurrent infections, particularly in atypical anatomic locations, in the absence of obvious secondary immunodeficient states (e.g. diabetes mellitus, corticosteroid use) should prompt an evaluation for underlying immunodeficiency, regardless of the age at presentation.

## Consent

Written informed consent was obtained from the patient for publication of this case report. A copy of the written consent is available for review by the Editor-in-Chief of this journal.

## Abbreviations

CGD: Chronic granulomatous disease; NADPH: Nicotinamide adenine dinucleotide phosphate.

## Competing interests

The authours report no competing interests with respect to this manuscript.

## Authors’ contributions

AGC: Drafted original version of manuscript, assisted with revisions. MMC: Manages the patient currently; critically reviewed and revised manuscript. AKE: Diagnosed the patient and treated while in hospital and as an outpatient for ~ 1yr, critically reviewed and revised manuscript. All authors read and approved the final manuscript.
